# Qualitatively exploring the intersection of health and housing needs in Canadian crowdfunding campaigns

**DOI:** 10.1186/s12889-022-12599-x

**Published:** 2022-01-26

**Authors:** Carly Doran, Valorie Crooks, Jeremy Snyder

**Affiliations:** 1 Department of Geography, Simon Fraser University, Burnaby, BC Canada; 2 Faculty of Health Sciences, Simon Fraser University, Burnaby, BC Canada

**Keywords:** Canada, Crowdfunding, Health, Housing, Health care, Classification scheme

## Abstract

**Background:**

Online crowdfunding platforms such as GoFundMe fundraise millions of dollars annually for campaigners. Medical crowdfunding is a very popular campaign type, with campaigners often requesting funds to cover basic health and medical care needs. Here we explore the ways that health needs intersect with housing needs in Canadian crowdfunding campaigns. In Canada, both health and housing needs may be addressed through legislative or policy intervention, are public health priorities, and are perceived as entitlements related to people’s basic human rights. We specifically develop a classification scheme of these intersections.

**Methods:**

We extensively reviewed Canadian crowdfunding campaigns on GoFundMe, the largest charitable crowdfunding platform, using a series of keywords to form the basis of the classification scheme. Through this process we identified five categories of intersection. We extracted 100 campaigns, 20 for each category, to ascertain the scope of these categories.

**Results:**

Five categories form the basis of the classification scheme: (1) instances of poor health creating the need to temporarily or permanently relocate to access care or treatment; (2) house modification funding requests to enhance mobility or otherwise meet some sort of health-related need; (3) campaigns posted by people with health needs who were not able to afford housing costs, which may be due to the cost of treatment or medication or the inability to work due to health status; (4) campaigns seeking funding to address dangerous or unhealthy housing that was negatively impacting health; and (5) people describing an ongoing cyclical relationship between health and housing need.

**Conclusions:**

This analysis demonstrates that health and housing needs intersect within the crowdfunding space. The findings reinforce the need to consider health and housing needs together as opposed to using a siloed approach to addressing these pressing social issues, while the classification scheme assist with articulating the breadth of what such co-consideration must include.

## Background

Crowdfunding platforms, such as GoFundMe, allow users to share their stories and appeal to wider social networks to collect donations to assist with meeting financial needs [1, 2]. While some people campaign to pursue dream projects, such as starting a business or taking an extended trip, others do so to request money to assist with their everyday financial needs, such as to cover the costs of food or rent. Unlike more established methods of fundraising, such as hosting local events or running charity drives or raffles, the money raised through crowdfunding can be requested by and given directly to the campaigner, which may facilitate ease of access [3]. While traditionally fundraising was often associated with exceptionality, such as to assist families with coping with extensive medical expenses or to help a person recover financially following a devastating house fire, the accessibility of online crowdfunding platforms to campaigners and visibility in social media spaces has made such fundraising for everyday expenses and basic necessities commonplace [4].

Crowdfunding to cover health or medical needs, often referred to as ‘medical crowdfunding’, is growing in popularity. This is especially true on the GoFundMe platform, where medical crowdfunding brings in the largest donation stream [4]. While this may not be unexpected in countries or jurisdictions that lack public health care coverage and thus where funding one’s care is thought of as a personal responsibility, research has shown that medical crowdfunding is growing in popularity in Canada and the United Kingdom where publicly funded health care is available [5, 6]. People campaign to assist with raising funds for a multitude of expenses related to medical care and health management, such as funding surgery abroad, paying for experimental treatments, covering prescription costs, as well as the expenses associated with getting to-and-from medical facilities [7, 8]. Given the frequency with which crowdfunding is used to campaign for medical and health-related expenses, it is not surprising to see the rise of research exploring medical crowdfunding and its associated trends, social dynamics, and ethical challenges [9–17]. This research has assisted with establishing a foundational knowledge base about medical crowdfunding, including how and why it is being used in particular countries such as the United States [18], Canada [14], the United Kingdom [9, 13], and China [19].

It is certainly likely that people who are unable to meet their own medical care and health needs are going to experience other financial challenges [1]. In the current analysis we work from this understanding to explore the intersection of health and housing needs in Canadians’ crowdfunding campaigns. By ‘intersection’ we are referring to *both* housing and health needs being articulated in a single crowdfunding campaign. We focus on this intersection in the Canadian context because it is acknowledged that both access to housing and health care are basic human rights nationally, via the National Housing Strategy Act’s right to housing amendments and the Canada Health Act, with legislative measures and national/provincial/territorial/regional/municipal initiatives to support achieving each [20, 21]. Focusing solely on Canada in this analysis thus allows for some consistency in the policy context. Meanwhile, it is recognized that there are gaps within both the housing and health care systems that leave some Canadians vulnerable to experiencing inadequate housing or homelessness and also inadequate or inequitable access to health care [22, 23]. Existing research has documented that medical crowdfunding by Canadians assists with documenting gaps in the health and social care systems [14], and it is reasonable to expect the same is true for housing supports. Further to this, having access to affordable housing and health care are both health determinants that are championed by Canadian public health agencies and practitioners [20, 24]. This is another important interconnection between the domains of health and housing, the intersections of which we explore herein.

Crowdfunding campaigns can serve as a source of rich data for learning about both the practice of crowdfunding and about social phenomena [6, 18]. Researchers have drawn on these campaigns to examine issues as diverse as crowdfunding for specific surgeries [25–27], local health system gaps and deficiencies demonstrated by crowdfunding [14], and campaign credibility factors [28]. Here we contribute to this burgeoning area of scholarship by using campaign narratives to explore the intersection of health and housing among Canadian campaigners and specifically develop a classification scheme of campaign types within these categories. By narratives, we are referring to the text that campaigners write to post on a crowdfunding platform to explain and justify their financial need to potential donors. While these narratives are self-generated, crowdfunding platforms often offer suggestions for how to write content that may appeal to donors. For example, GoFundMe encourages campaigners to share personal details that appeal to readers’ emotions in their narratives and to supplement their narratives by adding photos and videos [4, 17]. Campaigns also include a title, a financial goal and donation tracker, and a place for donors to make comments. Further to this, some campaigners opt to include ongoing updates on their campaign and lives. While for this study we gathered the full content of campaigns we identified, including amounts requested and raised along with the number of donors, the current analysis focuses specifically on the content of the narratives. In the section that follows we detail our process of developing a classification scheme of the housing-health intersection in the Canadian crowdfunding landscape. We then move to explore the scope of the scheme, providing an in-depth exploration of each of the five categories of intersection. In our discussion we touch on the wider relevance of this analysis and the fact that the scheme underscores the importance of taking a non-siloed approach to understanding both health and housing needs in addition to offering directions for future research.

## Methods

GoFundMe is the most popularly used crowdfunding platform for personal health-related expenses [4, 29], and for this reason we opted to conduct this analysis using campaigns hosted by this site. To start, we developed a list of broad keywords (e.g., medical, health, Canada, house, housing, home) to search using GoFundMe’s search function that related to housing and health. Campaigns including these keywords were then restricted to campaigners located in Canada in order to select campaigns facing similar health and housing pressures. We then independently reviewed the titles of the campaigns that were generated through these initial searches in order to refine our keywords and begin to classify the different types of campaigns that dealt with both housing and health. Our process of developing and refining keywords and then word searching in a platform’s search function to develop a dataset is well established in the crowdfunding literature ^e.g.,^[6, 8, 11]. Following this, a team meeting was held to develop a more expansive list of keywords and to identify emerging campaign categories that should be part of the resulting classification scheme of housing and health intersections. Over the course of several weeks, from November 2019 to March 2020, GoFundMe searches took place, new keywords were determined, and the narratives of identified campaigns were reviewed on an iterative basis until the first two authors had developed a proposed classification scheme that characterized what emerged as the dominant intersections of housing and health among Canadian crowdfunding campaigners. Searches ceased when a series of 25 reviewed campaigns resulted in no new intersections being identified.

After a proposed classification scheme was created, all team members independently reviewed the narratives extracted from GoFundMe by the lead author of an assigned sample of 20 campaigns within each of the five category types that form the basis of the scheme. These 100 campaigns were organized in a shared spreadsheet that summarized key campaign details (e.g., title, date created, social media shares, campaigner location) and a link to the full campaign on GoFundMe. Following an independent review of campaign narratives, a team meeting was held to confirm the scope and scale of each category and the scheme as a whole. Agreement was reached about the framing of each of the five categories that form the classification scheme, including their distinctiveness and any interrelations between categories.

Following achieving confirmation among the investigators regarding the classification scheme, the lead author reviewed the narratives of each of the 100 previously extracted campaigns to ensure that all were assigned to the correct category. The lead and second authors also conducted another keyword search within GoFundMe to ensure that no types of campaigns situated at the intersection of housing and health written by Canadian campaigners had been missed. No new categories that warranted inclusion in the scheme were identified at this stage. The first author then extracted verbatim quotes that illustrated the scope of each of the categories from the narratives of the 100 campaigns captured in the spreadsheet. These quotes were independently reviewed by the team to achieve one final form of confirming interpretation of each category in the classification scheme. Consensus was reached regarding the interpretation of the extracts, after which operational definitions for each category were created.

We believe that our use of investigator triangulation throughout the process added considerably to the rigour of our qualitative study design through enhancing dependability [30]. Our detailed design process also enhanced the trustworthiness of the resulting classification [31]. In the findings section that follows we present the classification scheme, offering details on each of the five distinct categories that form the basis for understanding how health and housing intersect in the medical crowdfunding campaign space. To enhance qualitative confirmability [30, 31], we include verbatim quotes from the 100 campaigns we used to finalize the classification scheme throughout the findings section.

## Results

Canadian medical crowdfunding campaigners were found to be fundraising for a variety purposes that drew together both health and housing needs. Some campaigners sought to relocate closer to health care or treatment facilities, while others requested funds to cover the costs of rent or home modifications. It was not uncommon for campaigns situated at the intersection of housing and health need to also request funds to cover secondary expenses, such as food, travel, or furniture. Of the 100 campaigns we extracted to refine our classification scheme, 20 from each category, we found that the average funding request was CDN$26,637, ranging from CAD$5 to CAD$250,000. Campaigns originated from across the country; however, 77% of campaigns were posted with intended recipients in the populous provinces of British Columbia (*n*?=?25), Alberta (*n*?=?21), and Ontario (*n*?=?31). Eight campaigns emerged from Canada’s Atlantic provinces. None originated in Canada’s north.

Following the analytic process described above, we identified five categories of housing-and-health intersections that form the basis of a classification scheme that characterizes such crowdfunding by Canadians. First, there were instances of poor health that created a need to temporarily or permanently relocate in order to access treatment or care. Second, some campaigns focused on house modification funding requests to enhance mobility as a result of impairment or some other form of health need. Third, campaigns posted by people with health needs who were unable to afford their housing costs, often due to treatment or medication costs or an inability to have involvement in paid employment, were identified. Fourth, some Canadians crowdfunded because they were in dangerous or unhealthy housing that was negatively affecting their health. Finally, some campaigns in the housing-health nexus were posted by people who experienced a cyclical or ongoing relationship between poor housing causing poor health, or the reverse. In the section that follows we characterize the scope of each category forming the classification scheme, incorporating some direct quotes from the 100 campaigns we used to confirm the scheme to give voice to the issues at hand.

### Health status created need for housing relocation

A number of crowdfunding campaigns at the intersection of housing and health relayed requests for donations to assist someone in poor health in need of relocation (e.g. move closer to treatment centers, specialized clinics, and/or trusted physicians). Commonly, campaigns followed the narrative that the recipient required permanent relocation to a new city where housing costs were too high to access care or they required support to afford temporary housing in another city, province, or even country while maintaining their primary residence. With regard to the latter, it was not uncommon for campaigners to indicate that they would need to move between home and the treatment/care location several times throughout a designated period. For example, “*[child] and I will be in and out of Vancouver constantly; sometimes staying in the hospital, sometimes staying there as an outpatient… We are looking to raise funds in order to cover the costs associated with staying in Vancouver…and unexpected medical expenses.”* Some campaigns even requested assistance to fund housing, cost-of-living, and travel expenses for a caregiver or additional family members to relocate with them temporarily or permanently. This was especially the case with families where the intended recipient was a child with siblings. While most campaigns expressed that funds were needed “*to help cover the expenses associated with treatment*” or “*ensuring [recipient] has somewhere to stay*”, they also characterized the need to relocate permanently or temporarily as a “*burden*” or “*added [financial] stress.*”

### Health status or impairment created need for house modification(s)

Permanent or temporary changes in health status, the onset of impairment, and/or fluctuations in mobility all brought about needs for house modifications and prompted Canadians to post crowdfunding campaigns. Typically, these campaigns sought assistance to increase the accessibility or livability of a house through the installation of stairlifts, elevators, railings, track lift systems or retrofitted bathrooms or kitchens. In most instances, such modifications need to be paid for privately and can be quite costly. In some instances, Canadians may be able to have such expenses partially or fully covered by government grants or private insurance [32], but this is uncommon due to eligibility criteria or prohibitive wait lists. As one campaigner explained: “*The coverage by the province [*via* government support programs] is limited, and their home will need renovations to accommodate a child with new prosthetics*.” Reflected in this quote, it was not uncommon for these types of campaigns to be posted on behalf of a recipient in need of having access to such modifications out of a *“desire to be in their homes as long as possible*.” As one family member explained in a campaign, *“We would like to make it so that he has access to the bathroom from his room and for him to have a roll in shower.*” It was not uncommon for campaigns such as this one to express that home modifications were not only unaffordable to the person they are intended to benefit, but that the costs were prohibitive to the wider family and thus a crowdfunding campaign was established to reach out to wider networks.

### Health status contributed to housing unaffordability

Some campaigners were unable to work due to mental or physical impairment, fluctuating symptoms of chronic illness, recovering from an acute health event, or other aspects of their health status. In some cases, this resulted in a complete loss of income while in others it resulted in a changed income due to a new reliance on income support programs; in both instances, these were circumstances that made covering the costs of housing challenging. In other cases, campaigners were able to maintain employment, but new costs associated with managing their health (e.g., high out-of-pocket costs for prescriptions) resulted in a redistribution of money previously allocated for housing. These were all circumstances that drove people to crowdfund to seek funds to assist with covering housing costs that were otherwise unaffordable. As one campaigner explained: “…*I’m not physically, mentally and or emotionally able to work or seek new work for the time being*” due to their health. In some instances, people were looking for funds to assist with paying existing rent or mortgage costs, while in others they were looking for funds to help them find housing. Regarding the latter, it was not uncommon for people to be crowdfunding to obtain financial support following a move out of some form of institutional care context and into private housing. As one campaigner explained, “*The mental healthcare system, the housing system, the systems in place for seniors and the disabled have all fallen short of being able to help us.*” This campaigner went on to explain that income support programs do not provide enough funding to cover the costs of safe housing. Perhaps unsurprisingly, many of those crowdfunding because of housing unaffordability related to their health status or health needs explained that they needed to “*either pay rent and starve or eat and get evicted*.” This sense of having “*slipped through the cracks*” in terms of being able to have affordable housing while managing their health needs pushed campaigners to make the decision to crowdfund to assist with housing costs.

### Deleterious housing created or exacerbated poor health

Limited ventilation, inadequate heating, fire damage, exposed wiring, mold, asbestos exposure, leaks, and allergens were all aspects of the housing environment that had negatively affected the health of some campaigners. People turned to crowdfunding to seek financial assistance with remediation or to move. As one campaigner told:

*We emailed our landlord asking if he had ever had the house tested for mold, he feigned ignorance and did not come to check out the problem. At this time, we started getting chronically ill, experiencing all sorts of sickness: chronic cough, sore throat, itchy eyes, sinus congestion, brain fog, exhaustion, fatigue, depression, gastrointestinal problems, infections, anxiety, rashes and even clinically diagnosed pneumonia.*

These campaigners typically expressed a need to have a “*safe and healthy home”* or “*a place to call home*.” It was also very common to campaigners dealing with such circumstances to be renters who attributed housing deficits to landlord neglect, and who attributed remaining in substandard accommodations to challenging financial circumstances. In fact, campaigners often felt the need to explain why it was that they had not already moved or addressed the inadequacies themselves. “*The place I was living in for ten years, I found out had asbestos and potentially black mold,*” and “*finding affordable housing in our area took a lot longer than expected, due to an extreme housing shortage*” so “*after a while of living in the apartment we started to feel unwell*.” It was also not uncommon for these campaigners to report having existing chronic health conditions that were being exacerbated due to exposure to their housing environment, while there were also instances of people reporting the onset of new diagnoses they attributed to this exposure.

### Cyclical relationship between poor health and inadequate housing

A small number of campaigners reported an ongoing, almost cyclical in nature, relationship between having inadequate housing and poor health or managing health needs. These campaigns were characterized by long narratives that reported a history of dealing with both challenges over an extended period of time and a broad appeal for financial support. For example, one such campaigner was campaigning on behalf of a her 62-year-old mother who had been suddenly evicted from her home. The campaign recipient had to choose between living with a new roommate whose cat “*causes major ammonia issues and have left her with breathing troubles before*” or becoming homeless because she could not afford to live on her own. Further to this, the campaign recipient was diabetic and had cycled in and out of homelessness for the last few years. “*That [the onset of diabetes] started the cycle of poverty that my mother has fought to break for two years*.” As another campaigner explained, this “*cycle of poverty*” can negatively affect health: “*Being chronically ill, homeless, and poor is tough on my mental health. It’s a vicious cycle that’s hard to break out of when things go bad.*” These campaigners turned to the power of crowdfunding to assist them with helping to break this cycle through improving both their health and their housing.

## Discussion

In the current analysis we sought to create a classification scheme of crowdfunding requests situated at the intersection of health and housing needs. The scheme we shared in the previous section is structured around five categories: (1) health status having created a need for housing relocation; (2) health status or impairment having created a need for household modification(s); (3) health status having contributed to having poor or no housing; (4) deleterious housing having created or exacerbated poor health status; and (5) a cycle of poor health contributing to poor housing and the opposite. Table 1 provides an overview of the scheme and the scope of each of the categories. Overall, the campaigns we reviewed often referred to a lack of wider health and social care supports as driving them to seek individual solutions to these needs via crowdfunding. This common motivation for seeking funding from others is one of the interconnections between the categories within the classification scheme. There are other interconnections. For example, it was common for campaigners across all categories who were housed at the time of campaigning to express a desire to remain in their homes in whatever ways possible. This was even true in some, but not all, cases of people experiencing health problems brought on by housing deficiencies. Many requests in this category were for funds to remediate these deficiencies as opposed to finance relocation. In the remainder of this section, we consider the findings in light of existing research as well as housing and health policy priorities in the Canadian context.

**Table 1:** Classification Scheme Overview

Category	Scope	Types of Needs Articulated
Health status creates need for housing relocation	Campaigners who needed to relocate in order to be closer to health care facilities or to improve their health	Relocation to be closer to a hospital providing ongoing treatment; Maintaining regular residence while funding temporary relocation to access treatment
Health status or impairment creates need for housing modification(s)	Campaigners whose health status or impairment(s) necessitated housing modifications, typically to improve accessibility	Building ramps or wheelchair turnarounds; Installing roll-in showers or ceiling tracks
Health status contributes to poor or no housing	Campaigners who were unable to cover housing costs, attributing this at least in part to their health and the costs associated with its maintenance (e.g., prescriptions)	Assistance with paying for some or all of rent and housing-related costs such as utilities; Costs associated with acquiring housing (e.g., first month of rent, damage deposit)
Deleterious housing contributing to poor health status	Campaigners living in deleterious housing that was negatively affecting their health	Assistance with covering costs of black mold removal; Costs associated with remediating structurally unsound housing (e.g., moisture in floorboards/walls, exposed electrical sockets)
Cycle of poor health status contributing to poor housing and the opposite	Campaigners who reported experiencing a long-term connection between poor health exacerbating poor housing and the opposite	Support to pay rent due to the inability to work; Costs of rebuilding a life shaped by multiple traumas

Existing research has shown that the health and social housing systems in Canada are unable to meet the needs of all Canadians for a number of reasons, including funding limitations and eligibility requirements that can serve as access barriers [33–36]. The classification scheme presented here, which explores the intersections of health and housing crowdfunding requests, reinforces how interconnected health and housing needs actually are. Housing is widely recognized as an important social determinant of health [37] and so it is not surprising that campaigners identified ways their health was shaped by housing status. While in the Canadian context access to health care and access to affordable housing are both thought of as basic rights [21, 37, 38], the ways in which these rights are translated into policy and practice are through distinct systems that are often siloed and funded through separate mechanism. For example, it is only recently that family physicians, who are at the frontlines of Canadian health care provision, have even been guided on how to identify and support patients experiencing homelessness and precarious housing, including by directing them to appropriate housing services [39].

The classification scheme summarized in Table 1 illustrates how gaps in supports around health or housing can exacerbate needs related to the other. For example, the lack of a national pharmacare program [40] and presence of high out-of-pocket cost of prescriptions to manage some health conditions led some campaigners to struggle with meeting housing expenses after covering medication costs. In such instances they clearly articulated health and housing needs as being interrelated and turned to crowdfunding as a solution. Overall, the classification scheme illustrates the depth and breadth of ways in which health and housing needs are interconnected in the crowdfunding space, and thus it supports wider calls for more integrative approaches to addressing such needs [37, 38].

The scope of the campaigns reviewed to form the basis of, as well as confirm, the classification scheme presented herein did not touch on some of the most pressing housing needs in Canada that have significant health implications. Specifically, no campaigns were reviewed that sought funds to address people’s personal experiences of two of Canada’s most publicly acknowledged housing crises: homelessness, particularly within major urban centres, and housing overcrowding in the North [12, 39, 41, 42]. While there were campaigns posted by people who were concerned about becoming homeless, none of the campaigns we reviewed were by those who identified as homeless. There were also no campaigns posted by people in Canada’s North, such as from campaigners in the North West Territories or Nunavut, let alone about overcrowding specifically. Overcrowding and homelessness and their implications for health can already fit within some or all the five categories of the classification scheme presented herein, and so we are not concerned that reviewing such campaigns would result in changing the scheme. Rather, the absence of campaigns posted by people experiencing homelessness or housing overcrowding in the North from the 200?+?campaigns reviewed to create and confirm this scheme likely speaks to the inequities inherent in the practice of crowdfunding. Although crowdfunding platforms often suggest that they provide equal opportunity to create campaigns and fundraise, many researchers have shown that the practice of crowdfunding actually reinforces inequities [14]. For example, people who do not have web literacy or reliable internet access, along with those who have limited personal networks or social capital, are disadvantaged when it comes to creating campaigns and/or reaching the fundraising goal [18, 43–47]. Considerable research has also shown that those engaged in crowdfunding may not be those who are most in need, equity-deserving, or vulnerable [3, 46]. This important to take into account when considering how much the health-housing need articulated in the crowdfunding space reflects the scope of needs at this intersection more widely.

Previously, scholars have contributed to the ongoing dialog surrounding the utility of crowdfunding platforms, by suggesting using this data to support policy making and assist with identifying support gaps [7, 13, 45]. By hand coding the ways in which users share their experiences with deleterious health and housing circumstances in campaign descriptions, our analysis supports that crowdfunding data can be impactful. Campaigners divulge their personal experiences and where they feel the greater systems are failing to provide them with the proper assistance to fulfill their basic needs, despite the resources already available to Canadians. This analysis provides a strategy to ideally reduce the number of campaigners who feel there is inadequate resources, by creating a classification scheme that highlights the importance of the relationship between specific individual needs to a specific set of circumstances. Additionally, this scheme can be utilized to help policy makers address gaps and strengthen our understanding of the connectivity between health and housing needs of Canadians.

Crowdfunding platforms have increasingly become a space for sharing stories, fueled by social networks and this analysis illustrates the ways in which these stories can be used to improve social systems. It is thus not surprising that there have been calls for policymakers to be attentive to crowdfunding platforms, trends, and content [48], especially because it has been shown that there may be a ‘substitution effect’ between medical crowdfunding and public health care coverage that policy officials should be attentive to [49]. While the findings offer a significant insight into the intersectionality of housing and health needs among Canadians, they also provide important direction for future research. For example, we have yet to know about the socio-economic details of those who campaign and how it could relate to a campaign’s financial success? How many details are required to be shared in order to collect donations? What is the relationship of those who donate to the campaigner? Furthermore, how can this analysis be deepened by looking at these needs in relation to the remaining social determinants of health such as education and literacy, income levels and healthy child development? In the past, scholars have argued to use crowdfunding data to identify emerging gaps in healthcare, therefore, through a variety of meaningful techniques, including discourse analysis in depth personal interviews it is possible to explore these questions and contribute to further research that focuses on preventative measures and risk discussion, avoiding these “gaps” all together. Our aim in undertaking this analysis is to better understand how health and housing needs are experienced by individuals who choose to use crowdfunding as a method to assist with their financial needs. The classification scheme created in this research could be further analyzed to help public health researchers properly address the existing Canadian housing crisis. Future researchers could use this classification scheme to better address the housing crisis and interview campaigners in order to find out where they felt the Canadian system failed them before turning to crowdfunding. In doing so, we hope for enhanced housing policy that supports the integration and of acknowledgement of individual health needs. We further aim for this analysis to serve as a model for future analyses using crowdfunding data to identify the gaps that exist within larger social systems.

## Conclusions

We set out to explore if and how and housing and health need, as characterized by Canadian crowdfunding, intersect in the domain of crowdfunding. Following an extensive review of campaigns, we identified five ways in which such intersections emerge. First, there were those whose campaigns indicated that their health status created a need temporarily or permanently relocate, and thus find new housing, as a result of their health status. Second, some campaigners requested funds to assist them with modifying or renovating their housing to meet a health-related need. Third, some campaigners requested funds to assist them with meeting their ongoing housing costs because they were no longer able to do so either due to not being able to work because of their health or the costs of treatments and medications. Fourth, some campaigners sought funding to assist them with addressing unhealthy or dangerous housing that was negatively affecting their health. Finally, there was a final group of campaigners who characterized a cyclical relationship between housing and health needs, where one informed or impacted upon the other in an ongoing fashion. Overall, the classification scheme we presented herein that characterized these five intersections, see Table 1 for a full synthesis, helps adds to existing research pointing to interrelationships between health and housing need through adding considerable nuance our understanding of the breadth of intersections that exist.

By revealing nuances in the interrelationships between health and housing need, this analysis helps to demonstrate the relevance of calls to look to crowdfunding campaigns as a promising source of detailed information about health and social care needs [14, 15]. However, in doing so we acknowledge that there are inequities inherent in the practice of crowdfunding that result in this practice being inaccessible to many of the most structurally vulnerable groups [2, 18]. For example, as we previously noted, we did not note any campaigns posted by those who identified as homeless. As a result, it is important to couple insights emerging from crowdfunding campaigns with those emerging from other sources in order to inform policy change or intervention. This holds true for the current analysis.

## Data Availability

The datasets used and/or analysed for this study (i.e., a list of the 100 campaigns reviewed to confirm the scheme) are available from the corresponding author.

## Abbreviation

GFM: Go Fund Me
